# Surgical and Radiological Anatomy of the Medial Patellofemoral Ligament: A Magnetic Resonance Imaging and Cadaveric Study

**DOI:** 10.3390/diagnostics11112076

**Published:** 2021-11-10

**Authors:** Vasileios Raoulis, Apostolos Fyllos, Michail E. Klontzas, Dimitrios Chytas, Vasileios Mitrousias, Konstantinos Banios, Thomas G. Maris, Apostolos H. Karantanas, Aristeidis Zibis

**Affiliations:** 1 Laboratory of Anatomy, Department of Medicine, School of Health Sciences, University of Thessaly, 3 University Str., Biopolis, 41110 Larissa, Greece; v_raoulis@yahoo.gr (V.R.); apofyl@hotmail.com (A.F.); 2 Department of Radiology, Medical School, University of Crete, 71500 Heraklion, Greece; miklontzas@gmail.com (M.E.K.); akarantanas@gmail.com (A.H.K.); 3 Department of Medical Imaging, University Hospital of Heraklion, 71110 Heraklion, Greece; 4 Department of Physiotherapy, University of Peloponnese, 20 Plateon Str., 23100 Sparta, Greece; dimitrioschytas@gmail.com; 5 Department of Orthopedic Surgery, University Hospital of Larissa, 3 University Str., Biopolis, 41110 Larissa, Greece; vasileiosmitrousias@gmail.com; 6 Department of Orthopedic Surgery, General Hospital of Karditsa, Peripheral Road Karditsa-Kastania, 43100 Karditsa, Greece; konmpanios@hotmail.com; 7 Department of Medical Physics, School of Medicine, University of Crete, 71500 Heraklion, Greece; tmaris@med.uoc.gr

**Keywords:** medial patellofemoral ligament, magnetic resonance imaging, surgical anatomy, radiological anatomy, dissection technique, cadavers/knee joint

## Abstract

The purpose of this study was to compare the measurement of several anatomical features of the medial patellofemoral ligament (MPFL) between magnetic resonance imaging (MRI) and by direct fashion during dissection. We hypothesized that the measurements between these two techniques would agree. MRI of 30 fresh-frozen cadaveric knees was followed by dissection. MPFL patella and femoral attachment were evaluated; their shape, length, and width were measured; and measurements were compared. MRI was deemed unreliable for the determination of several of the aforementioned anatomical features. Important findings include: (a) observations on MPFL attachment at medial patella side and attachment to quadriceps were identical between dissection and MRI; (b) average width at patella insertion was significantly different between the two methods (*p* = 0.002); and (c) an attachment to the quadriceps tendon was present in 20/30 specimens and d. detailed measurements of a thin, non-linear, and three-dimensional structure, such as the MPFL, cannot be performed on MRI, due to technical difficulties. This anatomical radiological study highlights the shape, anatomical measurements (length and width), and attachment of the MPFL using a relatively large cadaveric sample and suggests that MRI is not reliable for detailed imaging of its three-dimensional anatomy.

## 1. Introduction

The medial patellofemoral ligament (MPFL) is the primary restraint to lateral patellar translation, contributing 50% to 70% of the total restraining force [1,2]. The MPFL is almost always ruptured after a lateral patellar dislocation [3,4]. The first episode of patellar dislocation may be treated nonoperatively; however, a redislocation is reported in up to 35% to 50% of patients [5,6,7,8]. MPFL reconstruction is a surgical option in patients with recurrent dislocations and is currently the first-choice procedure for patients after more than one or two episodes of patellar dislocation [9,10,11].

A thorough understanding of the native anatomy and function of the MPFL is necessary in order to achieve a successful ligament reconstruction. The MPFL is anatomically a variable structure, which is located in a layer below the vastus medialis muscle. It has insertions at variable levels of the medial femoral epicondyle and medial edge of the patella [12,13].

The purpose of this study was to compare the measurement of several anatomical features of the MPFL between MRI and by direct fashion during dissection. We hypothesized that the measurements between these two techniques would agree.

## 2. Materials and Methods

This study was approved by the IRB (Institutional Review Board) of the Medical School of University of Thessaly as part of the PhD thesis of one of the authors (ID number 2754). A total of 30 fresh-frozen cadaveric knees (18 male, 12 female; mean age, 65.2 ± 8.0 years) were obtained through an Anatomy Donation Program and stored at −21 °C. The specimens were thawed for 24 h before MRI measurements and the dissection experiment at room temperature (18°). There was no medical history of bone or soft tissue injury, surgery, or osteoporosis in any of the 30 fresh-frozen knee cadavers.

### 2.1. MR Imaging Protocol

Before dissection, MRI was performed on all specimens using a high-resolution 3D T1-w Volumetric Interpolated Breath-hold Examination (VIBE) sequence, which enabled a slice thickness of 0.6 mm. The specifications of this high-resolution 3D sequence are presented in Table 1. Images were analyzed on an Evorad RIS-PACS system (Evorad, Athens, GR).

### 2.2. Dissection Technique

**Figure 1:**
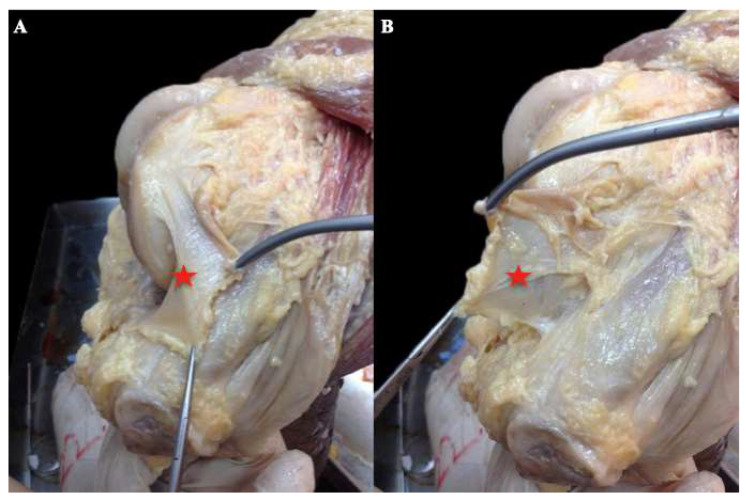
(**A**,**B**): Cadaveric right knee, medial side. The patella has been reflected medially. The capsule (red star) is detached from the second layer.

Midline incision was performed in every cadaver knee with knee flexion at 90°, detaching skin from the subcutaneous fascia and exposing the front side of the quadriceps–patella–patella tendon complex. Afterwards, the knee joint was exposed via a lateral parapatellar incision. The patella was consequently reflected medially, revealing the medial capsule. The third layer was detached, isolating the synovial capsule (Figure 1). In this way, the second layer was reached quickly and safely. The fibers of the MPFL were identified by palpation and direct vision and marked with pins. The patella was then reflected back to its original position. Finally, the first layer was detached from the superficial to deep tissues, in order to dissect and visualize the superficial surface of the MPFL. During the conceptualization of the project, extreme adhesions were observed between the first and second layer, making dissection form superficial to deep extremely difficult and placing the integrity of the MPFL at risk.

Measurements performed during dissection:

1. Average maximal length of MPFL
2. Average width of MPFL at three different sites: femoral and patellar insertion, mid-length.
3. Location of the femoral attachment relative to the medial epicondyle and the adductor tubercle
4. MPFL attachment at the medial patella side was determined by dividing the patella medial side into three equal parts (proximal, middle, and distal).

Other anatomical features also documented during dissection:

5. Whether there was quadricep attachment of the MPFL
6. Shape of the MPFL (whether it was triangular or not)
7. Thickness of the MPFL

### 2.3. Interobserver Agreement

Interobserver agreement was tested between two equally-experienced orthopedic surgeons for the dissection experiment for all measurements. Each specimen was measured by two different investigators, using the same Vernier caliper (accuracy 0.01 mm). MRI measurements were performed by two equally experienced radiologists. Interobserver agreement for measurements was tested for all measurements. Prior to the actual agreement study, consensus was reached on the measurement protocol. Bias due to differences of equipment (e.g., different screen size and analysis) was eliminated by using the same radiologist equipment and the same Vernier caliper for cadaver measurements. Each observer was blinded to the other observer’s measurements for the interobserver agreement analysis.

### 2.4. MRI Measurements

**Figure 2:**
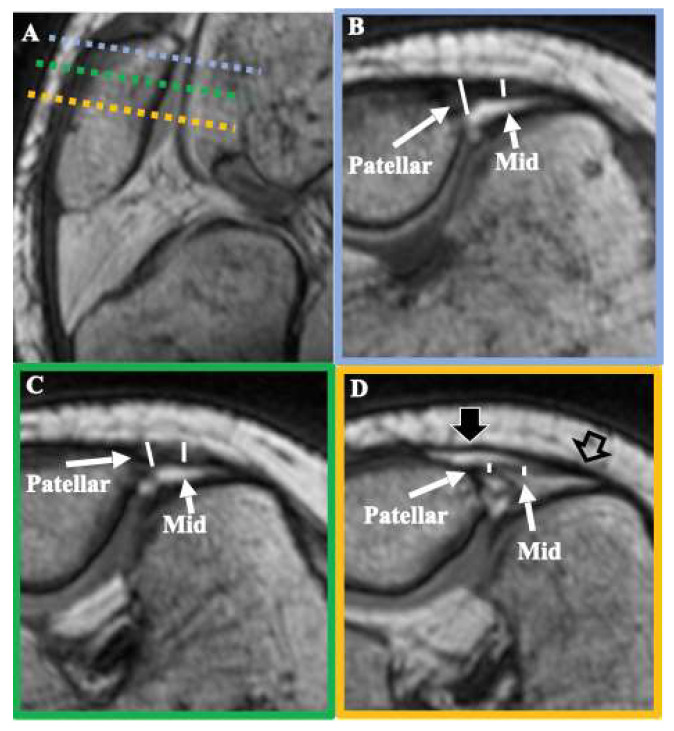
T1 3D VIBE images of a cadaveric knee demonstrating MPFL thickness measurements. Image (**A**) demonstrates the oblique axial planes where MPFL thickness measurements are collected, where dashed colored lines represent the oblique axial plane indicated by the respective frame color of images (**B**–**D**). Thickness measurements at the patellar attachment and the middle (Mid) of the MPFL are presented with white lines on a proximal (**B**), middle (**C**), and distal (**D**) point of the ligament. Distally, the surface layer of the joint capsule (thick black arrow) can be differentiated from the deep layer of the MPFL. However, it is evident that the capsule merges with the MPFL at the femoral attachment site (thick empty arrow), rendering unreliable the measurement of the exact MPFL femoral insertion area and thickness close to the femur.

During the initial design of the study, we observed that the aforementioned seven measurements could not be accurately performed on MRI, due to technical limitations. The length of MPFL had been measured in the past using the technique described by Higuchi et al. [14]. This technique was developed using low-quality images from open MRI, where it is impossible to discriminate between the MPFL, the MCL, and adductor magnus tendon and the capsule at the point where they contact with the femur. Furthermore, in our study, even though the examinations were performed using a powerful custom 1.5 T system reinforced with 3 T gradients, which enabled superb spatial resolution using closed MRI equipment, it was still impossible to demarcate the exact point of attachment of the MPFL to the femur. Consequently, length measurements on MRI were considered misleading and were avoided for the sake of accuracy. Therefore, even with the use of multiplanar reconstruction and curved MPR, the following measurements cannot be accurately recorded with the use of MRI: average maximal length, average width at femoral insertion and mid-length of the MPFL, location of the femoral attachment relative to the medial epicondyle and the adductor tubercle, and thickness and shape of MPFL (Figure 2).

The following were ultimately measured: average width at patella insertion, patella third of MPFL attachment (proximal, middle, distal, or combination of the above).

### 2.5. Statistical Analysis

Average width measurements at patella insertion were compared between dissection and MRI with the Students’ T-test for paired samples. Statistical significance was set at 0.05. Intraclass correlation coefficient (ICC) was used to determine interobserver agreement. Interpretation of ICC values was performed as proposed by Fleiss, with ICC values <0.4 considered a poor agreement; 0.40–0.74 fair to good agreement, and >0.75 excellent agreement, beyond chance [15]. All statistical analyses were performed in SPSS v21.

## 3. Results

The MPFL was found intact in all fresh-frozen cadaveric knees. The MPFL was located at the second layer of the medial part of the knee, as a complete ligament.

### 3.1. Measurements Performed during Dissection

**Figure 3:**
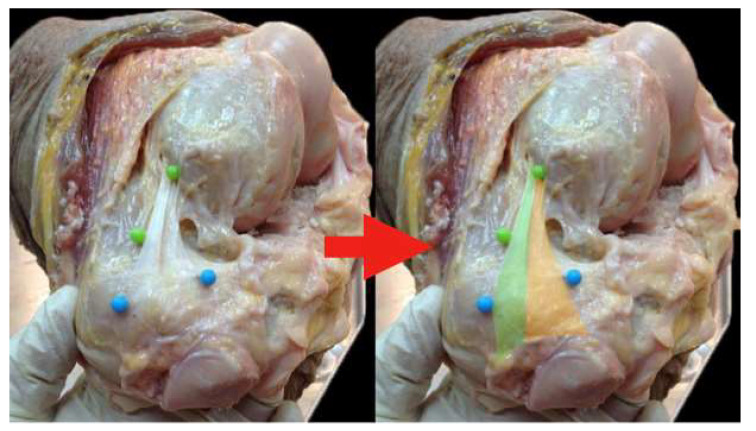
Left knee, anteromedial side. The MPFL shape is highlighted with color on the left part of the photo (green and orange). The green part represents the part of the MPFL that ultimately attaches at the quadriceps tendon, whereas the orange part represents that part of the MPFL inserting at the upper half of the patella.

**Figure 4:**
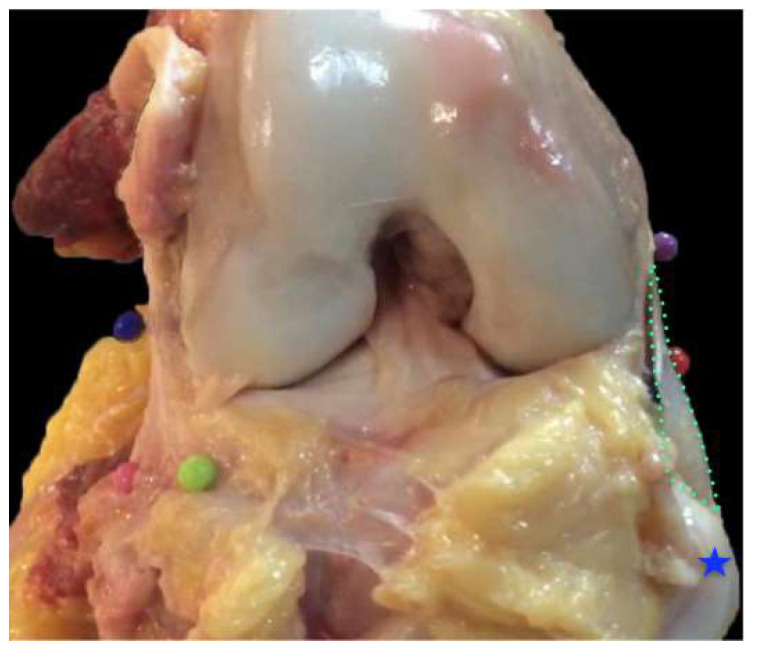
Right knee, anterior side. The patella (blue star) has been reflected. The MPFL (and its width) is depicted inside the green dotted line.

1. Average maximal length of MPFL: 60 mm (range 55–64, SD 8)
2. Average width: femoral insertion 6.8 mm (range 5.7–7.2, SD 2.2), middle insertion 11 mm (range 10.5–13.2, SD 2), and patella insertion 28.8 mm (range 25.1–34.3, SD 3)
3. The MPFL femoral insertion was found at 7 mm (SD 2.4) proximal and 7.1 mm (SD 3.6) posterior to the medial femoral epicondyle. In relation to the adductor tubercle, it was located 9 mm (SD 2.1) distally and 1.2 mm (SD 2) anteriorly.
4. The proximal third of the patella was always involved in MPFL attachment (30/30 specimens), compared to 70% (21/30) of the middle third and 10% (3/30) of the distal third
5. An attachment to the quadriceps was present in 20/30 specimens (Figure 3)
6. Its shape was consistently triangular (“the sail of a sailboat”) in all specimens
7. Mean thickness was found to be 0.91 mm (range 0.79–1.3, SD 0.15) (Figure 4)

### 3.2. Measurements Performed on MRI

Average width at patella insertion: 30.9 mm (range 22–40, SD 5.1)

1. The proximal third of the patella was always involved in MPFL attachment (30/30 specimens), compared to 70% (21/30) of the middle third and 10% (3/30) of the distal third
2. An attachment to the quadriceps was present in 20/30 specimens

Observations on MPFL attachment at the medial patella side and the attachment to quadriceps were identical between dissection and MRI. Average width at patella insertion was significantly different between the two methods (*p* = 0.002)**.**

### 3.3. Interobserver Agreement

For the dissection, the interobserver agreement was excellent. ICC was measured as 0.98, with 95% CI between 0.97 and 0.99.

For the MRI, the interobserver agreement was excellent. ICC was measured as 0.89, with 95% CI between 0.85 and 0.9.

## 4. Discussion

The primary finding of this anatomical and radiological study is that detailed measurements of a thin, non-linear, and three-dimensional structure such as the MPFL cannot be performed on MRI. A secondary finding was the identification and agreement of the broad attachment of the MPFL on the patella site between dissection and MRI. The rate of its insertion in the quadriceps was in accordance with most recent studies and reviews in the international literature [16,17,18]. Additionally, there was confirmation of the consistency of MPFL shape. Kang’s theory of two separate bundles, each with different functions, could not be confirmed macroscopically [19]. This anatomical study was the first to use a large number of cadaveric specimens (30 fresh frozen knees).

Even though there was agreement in the qualitative data on patella insertion of MPFL between dissection and MRI, quantitative measurements of width were significantly different. From an anatomical and clinical point of view, the wide insertion on the medial side of the patella, as highlighted in our study, confirms the importance of an anatomical reconstruction of the MPFL with double-bundle techniques. Other biomechanical studies have proven that reconstruction with a single bundle does not reproduce the complex shape of the MPFL and could lead to a possible rotation of the patella during knee flexion [20,21,22]. It should be mentioned though, that the high-resolution sequence used for MRI examinations (0.6 mm) provides a spatial detail that can demonstrate the ligament with the finest detail provided in any MRI study of MPFL to date.

Kang et al. described the MPFL as two separate bundles merging with a common origin [19]. The authors used the term “lower straight bundle” to describe the attachment fibers to the medial lateral part of the patella and “upper oblique bundle” for the fibers of the second bundle, which are attached to the quadriceps tendon and the upper medial part of the patella. The authors reported the different functions of these fibers, the lower bundle acts as a static stabilizer and the upper bundle as both a static and dynamic patella stabilizer. In our study, the macroscopic appearance did not resemble two separate bundles, but rather a single fan-shaped ligamentous structure. Furthermore, during dissection, a quadricep extension of the patella insertion was found in 21 knees (70% of the sample). This was also confirmed during the MR measurements. Previously, Fulkerson and Edgar presented these fibers of the MPFL as a distinct ligament, the “medial quadriceps tendon femoral ligament” [17]. This term describes the specific fibers that attach to the quadriceps tendon. Other studies have included these fibers as variable components of the MPFL that do not necessarily form a distinct ligament [19,23]. Owing to this, the entire ligament has also been referred to as the “medial patellofemoral complex”.

The preparation was held from the inside of the joint because during the pilot anatomical preparations, we found that it was easier to access the MPFL without injury, as the third layer is less attached to the second layer than the first, and as soon as the capsule was detached we came into direct contact with the MPFL, either by direct vision or by palpation [24].

Femoral insertion was much discussed in the first anatomical studies between the 1990s and mid-2000s, and was briefly described as an attachment directly into the adductor tubercle or the medial femoral epicondyle. Later studies located the attachment in an area between the medial epicondyle and the adductor tubercle, named “Nomura’s point” [4,25]. According to the present anatomical study, the femoral insertion is located in a separate location from both the adductor tubercle and the medial epicondyle, it occupies a concave area between these two osseous structures, with an average with of 6.8 mm. Therefore, our measurements could be taken into account during the femoral fixation of MPFL reconstructions. However, the precise determination of the femoral fixation area in order to avoid large incisions can be achieved with the help of intraoperative true lateral radiographs, as shown by Schöttle [26,27,28]. Our MRI findings support the notion that it is currently probably not possible to identify the femoral attachment of MPFL on MRI. As mentioned by Dirim et al., it is not possible to discern a potential attachment of MPFL to the tibial collateral ligament, since it is not possible to differentiate the capsular and ligamentous structures close to the femoral attachment of the MPFL [29]. This is the reason that accurate measurement of the length of the ligament is not feasible on MRI.

This study comes with methodological limitations. The age of the cadaveric knees created a limitation, since they came from an elderly population, not reflecting the younger population that usually require MPFL reconstruction. In addition, biometrics, such as the subjects’ height, patella size, and limb length, were not available for the cadavers. Anatomical differences between sexes were not taken into account. However, this anatomical study was the first to use such a large number of fresh cadavers (30 knees) that had not had previous surgery or suffered a major injury.

## 5. Conclusions

Given the importance of this structure to the stability of the patella, it should be reconstructed as anatomically as possible. This study highlights the MPFL as a separate ligamentous structure, in the shape of a sailboat, with a relatively wide footprint between the medial femoral epicondyle and the adductor tubercle. Its patella insertion varies, it is most commonly found in the proximal half of the medial side of the patella, while in several cases it inserts on the medial side of the quadriceps tendon.

## Figures and Tables

**Table 1:** MRI protocol.

	1.5-T MR Scanner, 4 Channel (Slew Rate: 200 mT m^−1^s^−1^)
High-resolution T1-w 3D VIBE	TR = 9.36 ms; TE = 3.52 ms; FOV = 18.3 × 22 cm; ST = 0.6 mm
